# Gallium Oxide Nanostructures: A Review of Synthesis, Properties and Applications

**DOI:** 10.3390/nano12122061

**Published:** 2022-06-15

**Authors:** Nishant Singh Jamwal, Amirkianoosh Kiani

**Affiliations:** 1 Silicon Hall: Micro/Nano Manufacturing Facility, Faculty of Engineering and Applied Science, Ontario Tech University, 2000 Simcoe St N, Oshawa, ON L1G 0C5, Canada; nishantsingh.jamwal@ontariotechu.ca; 2 Department of Mechanical and Manufacturing Engineering (MME), Ontario Tech University, 2000 Simcoe St N, Oshawa, ON L1G0C5, Canada

**Keywords:** gallium oxide, nanowire, nanostructures, nanofabrication methods, optoelectronics, optical materials, semiconductor

## Abstract

Gallium oxide, as an emerging semiconductor, has attracted a lot of attention among researchers due to its high band gap (4.8 eV) and a high critical field with the value of 8 MV/cm. This paper presents a review on different chemical and physical techniques for synthesis of nanostructured β-gallium oxide, as well as its properties and applications. The polymorphs of Ga_2_O_3_ are highlighted and discussed along with their transformation state to β-Ga_2_O_3_. Different processes of synthesis of thin films, nanostructures and bulk gallium oxide are reviewed. The electrical and optical properties of β-gallium oxide are also highlighted, based on the synthesis methods, and the techniques for tuning its optical and electrical properties compared. Based on this information, the current, and the possible future, applications for β-Ga_2_O_3_ nanostructures are discussed.

## 1. Introduction

Gallium oxide is an ultra-wide band gap semiconductor material. The band gap of gallium oxide is 4.8 eV, which is far greater than the band gap of other semiconductor materials, like Silicon (1.1 eV), SiC (3.26 eV) and GaN (3.4 eV) [1,2,3]. This semiconductor material is also a Transparent Conductive Oxide (TCO), which gives gallium oxide another advantage over the other materials, like silicon, in solar cell application. The electrical conductivity of gallium oxide is high, which allows the material to perform better than GaN and SiC in terms of low resistance electrical contacts. This is due to the presence of point defects in the structure of gallium oxide [4,5]. Gallium is an unstable and rare element in nature. Ga_2_O_3_ is soluble in most acids and alkalis; however, it is stable and insoluble in water. Ga_2_O_3_ has five different atomic structures (α-, β-, γ-, δ-, ε-); among these structures β-Ga_2_O_3_ is the most stable one [6]. This has piqued the interest of researchers in the beta polymorph.

Gallium was discovered in the 19th century. However, it was in the 1960s when researchers started researching the various structures of gallium oxide. In 2000, the focus moved towards structural analysis of gallium oxide using the arc discharge method [7]. The arc discharge method was used by many researchers, mainly for the synthesis of monoclinic nanowires of Ga_2_O_3_ [8,9,10,11].

**Figure 1:**
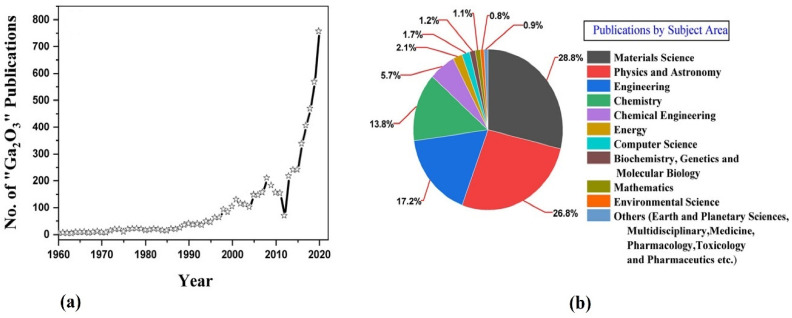
(**a**) the number of publications of gallium oxide over the years from 1960 to 2020. (**b**) the number of publications based on subject area [21]. “Reprinted with permission from Ref. [21]. Copyright (2017), Elsevier”.

The world is moving toward options for sustainable energy sources, and one of the most prominent renewables is solar power [12,13]. Solar energy is abundant; however, collecting solar energy is challenging due to the band gap of the material used to make the cells. Ga_2_O_3_ is a good candidate to be of prominent use, as the higher band gap would allow for a collection at varied wavelengths across a deeper ultraviolet range [14,15,16]. The research on gallium oxide is getting more and more recognition (Figure 1), as researchers have started to realize its potential in solar cell and photovoltaic devices [17,18]. Although devices based on gallium oxide are only n-type or unipolar, there is a possibility that p-type doping of β-Ga_2_O_3_ could pave a path for deep UV-optoelectronic devices and applications relating to high power [19,20].

Gallium oxide can be effectively used as a sensor for detecting gases, including carbon monoxide and hydrogen; also, it can be used as an oxygen sensor if there are sufficient oxygen vacancies available [22,23,24,25,26]. β-Ga_2_O_3_ has oxygen vacancies present in the interstitial sites and this allows the absorbance of gas, due to which there is a change in the resistivity of the material. It is challenging to produce materials appropriate for gas sensing, as several factors need to be considered: the synthesis processes need to be appropriately selected, and the process parameters must be defined based on desirable sensing and structures. Selection of suitable synthesis process and parameters is necessary, as varying the parameters or process can result in a varying Ga/O ratio, which can directly affect properties [27]. The formation of the semiconductor is dependent on the parameters, which can change the vacancies and limit the use. The various applications for a material depend on the factors that affect its synthesis and properties. For instance, the doping and type of semiconductor material affects the intended application [28,29,30].

Silicon is a semiconductor material for power electronic applications, widely used since the 1950s [31,32,33,34]. The major advantage of silicon is its abundance [35]. However, silicon cannot be used for higher voltages, due to its small band gap. For high-power applications, silicon becomes less reliable, and, thus, SiC and GaN are employed, having band gaps of 3.26 and 3.4 eV, respectively [36,37,38,39,40]. Moore’s law, which talks about the doubling of transistors in an IC circuit every 2 years with the price of the devices being reduced to half, is also another factor. Advances in technology require more in smaller size, which makes it difficult for silicon with its limited properties. Consequently, gallium oxide could cover areas where silicon cannot be used, including in higher power applications, as it would allow reduced power losses and increased efficiency. The Baliga’s Figure of Merit (BFOM) is higher for gallium oxide when compared to others. This parameter is used to define the materials for high-power applications. Gallium oxide has various applications, and, due to its high luminescence property, it can be used as a drug carrier in the biomedical field [41,42,43,44,45]. This would allow the drug to be detected properly within human cells [42].

Furthermore, the aforementioned properties of gallium oxide can be enhanced upon when synthesizing nanostructures of the material. Choosing the correct and most efficient technique for the synthesis of gallium oxide nanostructures is an important task, as the properties of the material depend on the structure, and the structure depends on the techniques and parameters employed in its synthesis. A wide range of synthesis techniques have been reported by researchers for the fabrication of nanostructured gallium oxide. The methods used to produce gallium oxide are generally high energy-consuming, provide low yields, and produce impurities [46,47,48].

In this review article, we will discuss in detail the main fabrication techniques for β-gallium oxide nanostructures, their optical and electrical properties and both current and possible future applications of gallium oxide.

## 2. Structures of Gallium Oxide

**Figure 2:**
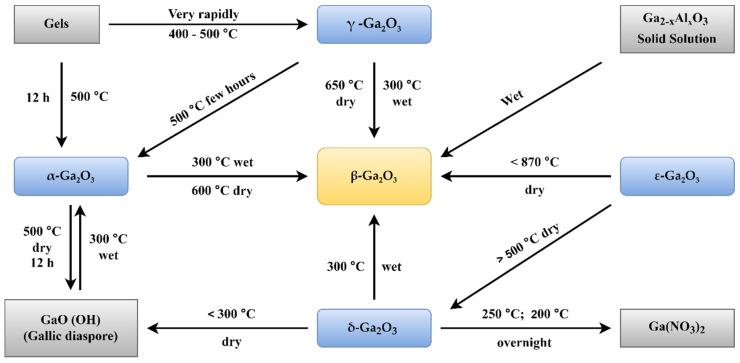
Transformation of Gallium oxide polymorphs to β-Ga_2_O_3_ [52]. “Reprinted with permission from Ref. [52]. Copyright (1952), ACS Publications”.

The arrangement of atoms in gallium oxide determines the different structures of gallium oxide, which are referred to as polymorphs (Table 1). For instance, α-Ga_2_O_3_(rhombohedral), β-Ga_2_O_3_ (monoclinic), γ-Ga_2_O (defective spinel), δ-Ga_2_O_3_ (cubic), ε-Ga_2_O_3_ (orthorhombic) [18,49,50,51]. The lattice parameter variations in the cell structures are responsible for these changes in the structure of the material. As all the structures of gallium oxide have 3D structures, the number of lattice parameters increases. This, in turn, increases the number of possible structures. However, all the structures are not stable. The α-Ga_2_O_3_ is a metastable structure apart from the stable beta structure. Most other polymorphs of gallium oxide (Table 1) transform to β-Ga_2_O_3_ at a temperature higher than 600 °C, which can be seen in Figure 2. Roy et al. [52] were the first to define all the polymorphs of gallium oxide in their work, as well as defining their stability.

**Figure 3:**
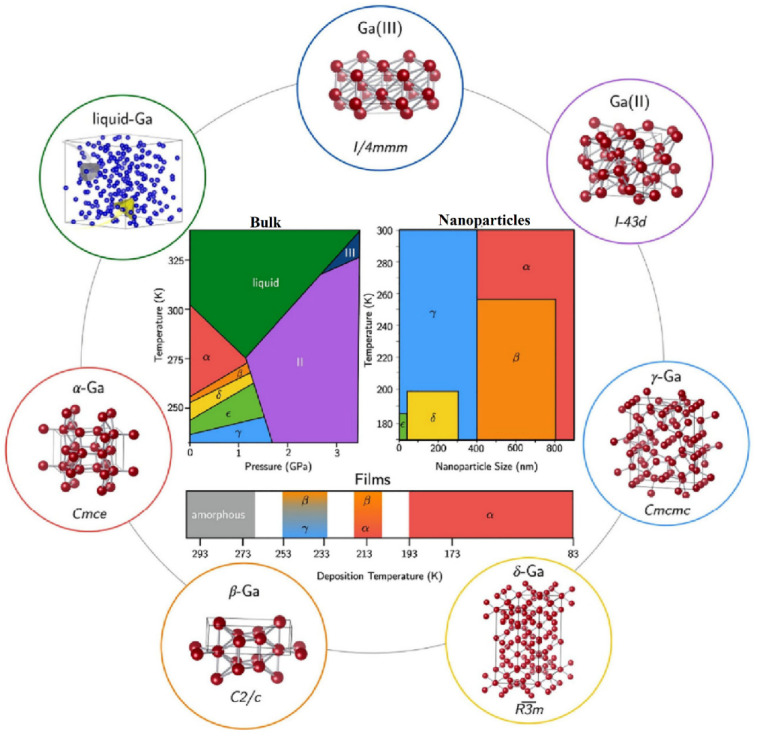
The various structures of gallium oxide and phase diagrams showing various phases at different pressures and temperatures [64]. “Reprinted with permission from Ref. [64]. Copyright (2020), De Gruyter”.

There is a transformation of β-Ga_2_O_3_ to α-phase, which occurs at higher pressure, and this change in the structure is irreversible (Figure 3). At higher pressures, α-Ga_2_O_3_ is most stable. The ε-phase of gallium oxide offers significant properties in the high-power application field. The transmission of this phase to the β-phase is at a temperature higher than 870 °C, which allows a space for devices to operate at high temperature. The stability offered by this phase allows it to have these properties. In a work done by M. García-Carrión et al. [63], it was observed that γ-Ga_2_O_3_ can be more efficient than the most popular beta phase for improving the carrier life of PEDOT: PSS in the application of hybrid solar cells. The produced gamma phase showed inter-band luminescence emission which was not reported earlier and, also, was not observed in the beta phase. This emission would allow the monitoring of any change in the band gap of the material with respect to photon energy.

## 3. Synthesis of Ga_2_O_3_

**Figure 4:**
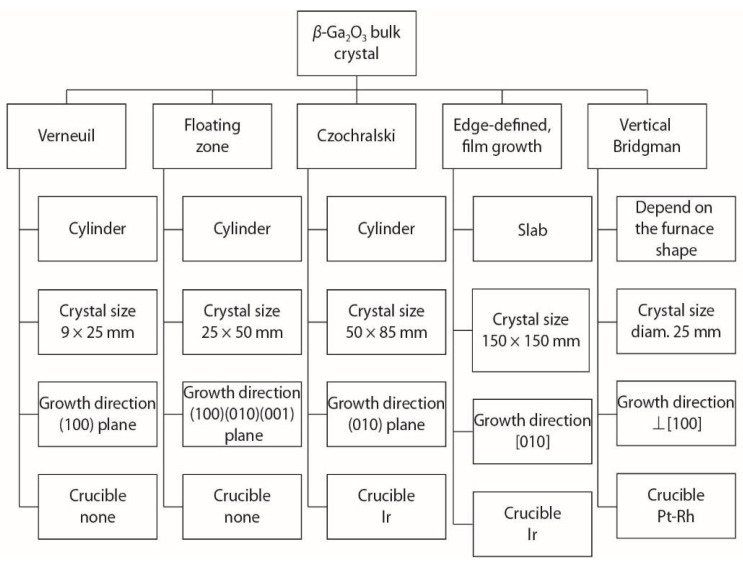
The various techniques for the growth of bulk β-Ga_2_O_3_ crystals [78]. “Reprinted with permission from Ref. [78]. Copyright (2019), IOP Science”.

Since the 1950s, there have been various techniques employed for the growth of bulk beta phase gallium oxide. Some of the reported techniques include the floating zone method (FZ) [2,65,66,67], the Czochralski method (CZ) [68,69,70], the Verneuil method [71,72], the edge-defined film fed growth method (EFG) [2,73,74] and the vertical Bridgman method [73,75,76]. These methods over time have been able to produce bulk β-Ga_2_O_3_ crystals in various crystallographic directions: (100), (010) and (001). EFG is the only synthesis process which makes n-type doping easier, enables large bulk crystals to be obtained and is the process having the highest growth rate [77]. Figure 4 highlights the various bulk crystal synthesis techniques for Ga_2_O_3_, including various shapes of the formed bulk material (cylindrical ingots or slabs). Crystal sizes are compared along with the growth direction planes, like 100, 010, 001. It can be noted that the floating zone and Verneuil methods are crucible-free methods, whereas, the Cz and EDFG methods require iridium (Ir) crucibles to contain the melt. The VB technique requires a platinum-rhodium (Pt-Rh) crucible [78].

### 3.1. Sol-Gel Method

Generally, the sol-gel fabrication method is one of the most convenient synthesis processes, as it is very flexible and does not require a complicated and expensive setup. It is also effective for materials with low curing temperatures [79]. The dip-coating method involves the coating of wafers on both sides using a polymer solution. Of these, Nafion is considered the best, as it is the strongest Lewis acid [80,81,82]. The basic method is to make a solution (or sol). This solution is then deposited over a substrate. This can be done by dip coating or spin coating. The next step is to dry or cure, which can be done by applying heat. Researchers used this method for gallium oxide in the middle of the first decade of the 21st century.

T. Miyata et al. [83] used the sol-gel technique to manufacture manganese-activated gallium oxide (Ga_2_O_3_:Mn) thin films for application in electroluminescent devices. Here, the solution was prepared by dissolving trimethoxy gallium and manganese chloride in methanol by constant stirring at room temperature in nitrogen gas for an hour; subsequently, water and hydrochloric acid were also added and then BaTiO_3_ sheets were immersed in the solution. The sheets were later dried and heated, and post-annealing was also performed. The films made from this process showed higher luminance, and post-annealing added an additional touch to the high luminance, which was observed above 500 and 100 cd/m^2^. The Ga_2_O_3_:Mn thin films were crystallized at a temperature of 800 °C, and an improvement took place at 1000 °C. The technique showed that the optical and electrical properties of the fabricated films were dependent on the annealing temperature. The comparison that the authors made with the thin films synthesized via sputtering and sol-gel techniques highlighted that the films prepared using sol-gel techniques showed better electro-luminance characteristics. This was primarily due to their better crystallization structures (fewer crystal defects). Furthermore, it was observed that heat treatment played an important role in obtaining better crystallized films.

In 2006, a group of researchers [84] prepared gallium oxide quantum dots, which were developed in silica gel as the matrix, using the sol-gel technique. The technique used two solutions to start with, the first being gallium oxide, where the gallium metal was added to the HNO¬3 while maintaining a pH value from 1-2. The other solution was SiO_2_ from its source, tetraethyl orthosilicate. The matrix was obtained by mixing the tetraethyl orthosilicate into a solution of water and ethanol, along with 0.1 N HCl for the purpose of hydrolysis, and stirring for an hour. Both parts were mixed and stirred for another hour while maintaining the predefined pH value all along and heating the solution to speed up the hydrolysis. Then, calcination at 200 °C followed for 4 h, followed by annealing at 400, 500 and 900 °C for an extended time of 11 h.

The process here had surprising results, as there were different molar ratios used for the Ga_2_O_3_:SiO_2_ (gallium oxide: silicon oxide). Previous studies had shown that only alpha phase gallium oxide could be made at temperatures below 500 °C [85,86]. However, the results after the XRD and HRTEM showed that it was possible to get the monoclinic beta phase of the gallium oxide at a temperature of 400 °C. This happened due to the diffusion of gallium nanoparticles into the matrix gel, which, in turn, began shrinking due to the heat and, thus, reducing the pores and decreasing the particle size [87,88]. Previous works have reported that to get a beta phase for gallium oxide, a temperature above 800 °C is required [89,90,91].

One study [86] mentioned that β-Ga_2_O_3_ could be obtained at an annealing temperature above 870 °C. However, in their technique of force hydrolysis, the solution aliquot of GaN, which had 0.0025 mol of gallium, was mixed with 90 mL water. It was then deionized and mixed for 15 min, followed by heating at 90 °C. Then, the aged portion underwent forced hydrolysis in a 100 mL screw-capped glass bottle at 90 °C. All this resulted in obtaining Ga_2_O_3_ at an annealing temperature of almost 500 °C.

### 3.2. Magnetron Sputtering

Magnetron sputtering is another popular process for the synthesis of gallium oxide employed by many researchers [92,93,94,95]. The process requires a chamber and an environment that would facilitate the formation of gallium oxide. Noble gas ions are used to bombard the target material and, due to this, there is a release of the particles from the material. These particles are deposited over a substrate. The term ‘target’ is used for the material to be removed and deposited on the substrate. Radio frequency is used to vary the potential of the electric current in the process, which results in better cleaning of the target and helps in more deposition [96,97]. The two major sputtering processes that are employed for beta gallium oxide are Rf-sputtering and magnetron sputtering. In most synthesis techniques that have been employed by researchers Rf-magnetron sputtering was employed.

An early experimental study [98] on the preparation of gallium oxide used RF magnetron sputtering. In this process, they prepared a thin Ga_2_O_3_ film with a 50 W power and an environment of argon gas. Several samples were prepared by changing a few parameters, such as the pressure for sputtering, temperature of the substrate, and the ratio of argon and oxygen gas, which was used as the environment for the process of sputtering. The samples were then annealed, with temperature constant at 1000 °C for an hour in air. While different parameters were changed, they observed that the annealing made the material more crystalline, and the other parameters, such as sputtering pressure, temperature, and gas ratio, really affected the formation of gallium oxide. The plasma that helped in deposition was greatly dependent on the ratio of the gas in the chamber. The intended application for the experiment was to use the gallium oxide for gas sensing; therefore, oxygen deficiency was beneficial for it. However, with the increased crystalline structure, the material would have a better conductive nature with more available vacancies [99]. The above study actually helped in noting how a change in the parameters of the sputtering process helps in the attaining of a different structure. The researchers here varied the sputtering pressure of argon gas in the first set of samples. Then, they varied the sputtering temperature in the second and followed by the ratio of oxygen and argon for the third set. A set of optimum findings were done to maintain the crystallinity of the thin film. The findings concluded that a lower gas pressure and a temperature at 200 °C optimized the crystalline quality. Lower oxygen was required for the process.

Nanostructure formation also has a similar nature. In 2011, research published in Vacuum vol. 85 [100] explored synthesized beta gallium oxide nanostructures on silicon as a substrate by annealing sputtering, using RF-magnetron sputtering. This synthesis was done in two phases. The first was to deposit the Ga_2_O_3_ and Mo (molybdenum) films on the substrate using the magnetron sputtering system. Acetone, isopropyl alcohol, and deionized water were used for the substrate prior to the process to clean it. The maintained pressure of the system was 0.00078 *Pa* along with passing of 2 *Pa* Argon gas. The power of the chamber was set at 150. The whole process was maintained at room temperature and the time period was 5 min and 90 min for Mo and Ga_2_O_3_, respectively. The second phase was where the deposited substrate was annealed, and nitrogen was passed first to create an ambient environment. Later, NH_3_ exposure was done inside the furnace at various temperatures of 850 °C, 900 °C, 950 °C and 1000 °C for 5 min. This process initially resulted in the formation of beta gallium oxide nanowires, and, then, at higher annealing temperatures, nanorods formed. At the final temperature, phase change (sublimation) of β-Ga_2_O_3_ was seen. Due to the passage of ammonia and nitrogen over the gallium oxide it underwent reaction and reduced and, in turn, gaseous gallium was produced, with the addition of a middle layer of Mo, which had excess O. As the annealing progressed, gallium oxide formed.

**Figure 5:**
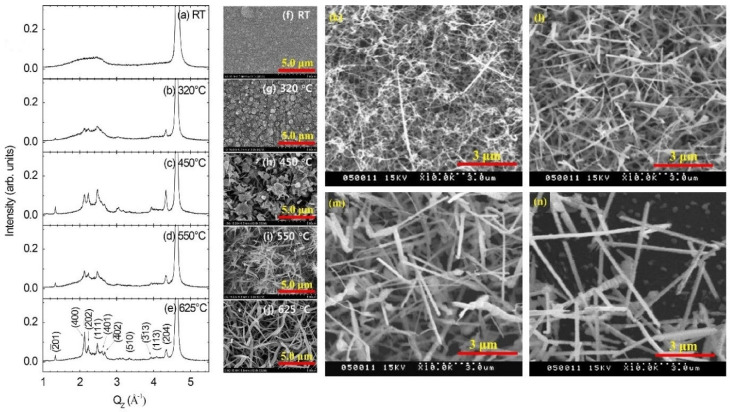
(**a**–**j**) XRD profiles along with the SEM images of the samples prepared by Lee et al., based on different substrate temperatures on SiN substrate. (**k**–**n**) the SEM images for the annealed samples [100]. “Reprinted with permission from Ref. [100]. Copyright (2011), Elsevier”. [101] “Reprinted with permission from Ref. [101]. Copyright (2015), Elsevier”.

A study in 2015 [101] used three different substrates: amorphous SiN/Si, SiOx/Si, and glass substrate for the deposition of β-Ga_2_O_3_ powder, using RF powder sputtering. This synthesis was done in a pure argon gas environment with a chamber pressure of 0.005 Torr, and the placement of the substrate was 4 cm away from the target. The power was set to 100 W. This sputtering was done at different substrate temperatures starting from room temperature to 625 °C. It was clear from the results of this process that the material for the substrate did not affect the formation of the nanostructures of beta gallium oxide. However, temperature played an important role here. The nanostructures at room temperature and at 320 °C were amorphous in nature, and crystal formation was seen at temperatures between 450 °C, 550 °C and 650 °C. The same phenomenon of growth was noticed with thin films and, later, the formation of nanowires. These formations are clearly visible in the SEM images in Figure 5a.

As has already been discussed, change in temperature of the substrate has an effect on the nanostructure formation (Table 2). This was an approach where an environment deficient in oxygen was used and through this a phase separation was achieved. The cluster formation of the metal was achieved from the stochiometric oxide of gallium. These clusters then helped in the formation of nanowires. The cluster formation was generally reported at lower growth temperature. Further, in 2021, a Si substrate of n-type quartz, using a 2-inch diameter beta gallium oxide wafer, was used [102] for the process. The deposition was done by normal RF magnetron sputtering. The temperature was maintained at room temperature, and the pressure was 0.005 Torr with a power of 50 W. Ar and oxygen were used as the gases for the environment in the chamber. The distance between the target and the substrate was 7 cm. Unlike the previous research, where annealing was not performed, the annealing temperatures employed in this process were 600, 800, 950 and 1000 °C. The lower annealing temperature resulted in a tendency for the formation of amorphous gallium oxide [103,104,105]. However, higher annealing temperature showed the monoclinic crystalline structure formation [106]. The XRD analysis of the sample in the paper showed that for higher temperatures, the value for intensity was higher, which was the trend for the samples observed, which corresponded to crystalline formulation at a higher temperature. Figure 5a–n clearly show that annealing the samples after the sputtering process yielded better nanostructures than heating the substrate while depositing the sputtered nanoparticles on the substrate.

The presented table shows how, at different annealing temperatures, different nanostructures were formed. It is clear that at a lower temperature and lower process power, the nanostructures are amorphous in nature, and as the temperature increases, the structures become crystalline at even lower power. However, at higher power and higher temperature, the sublimation of β-Ga_2_O_3_ can be observed. In general, published results show the annealing process plays an important role in gallium oxide formation; also, it was observed that combining of annealing and the addition of Mo with excess oxygen improved the formation of Ga_2_O_3_. The RF power has a minor role, as even with a lower power in [101] (when compared to [100]) there was formation of Ga_2_O_3_ nanowires.

### 3.3. Chemical Vapor Deposition

Chemical vapor deposition (CVD) is the process where the substrate is used for the deposition of nanomaterial in a vacuum chamber, and a specific deposition time is allotted [107,108,109,110]. Basically, it is an atomic layer deposition process. The layers are deposited on the surface, which is how the microfabrication process works. There are many methods in CVD, such as atomic pressure CVD [111,112], low-pressure CVD [113,114], and ultra-high vacuum CVD [115], which are based on the conditions under which the materials are synthesized. CVD can also be classified based on the parameters, such as whether a hot wall or a cold wall is used for the heating process of the substrate. For the preparation of gallium oxide, researchers have mostly employed a metallo-organic chemical vapor deposition (MOCVD) technique [116,117,118]. This technique is a modern improved CVD technique that is employed to produce a semiconducting thin film. Most researchers prefer this method, as it offers large control over the parameters that are specific for the growth of the nanostructures [119]. This process is comparable to low-pressure CVD. Here, gases carry the metallic portion, and the substrate is heated, which is where the decomposition takes place [120].

CVD, which is normally done in a vacuum chamber, can also be done using aerosol, which is typically called Aerosol Assisted Chemical Vapor Deposition (AACVD) [121,122,123]. This useful technique helps to increase the rate of deposition, as atmospheric pressure is used [124]. Another employed technique in CVD is Mist-CVD. This is a low scale technique, which is done in ambient conditions. It is an easier technique and it has often been reported regarding α-Ga_2_O_3_ structure [53,125,126]. There have been several authors who have used this for beta gallium oxide growth as well [127,128]. The working temperature for this technique is lower than for normal CVD.

**Figure 6:**
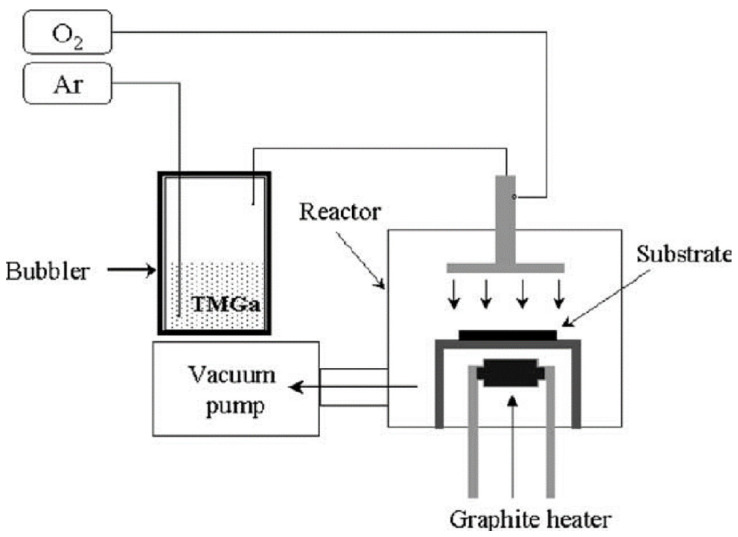
Schematic diagram of the MOCVD [129]. “Reprinted with permission from Ref. [129]. Copyright (2004), Elsevier”.

In their 2004 experimental work (Figure 6), Kim et al. [129] used a sapphire substrate with (0 0 0 1) orientation. A Trimethylgallium and oxygen mixture was used, and the flow rate for the oxygen was maintained at 6 sccm, with argon at a 30 sccm flow rate. The temperature of the substrate was set at 600 °C, and the time for deposition was set to 5 min. Structural analysis was done on the samples using SEM and TEM, and photoluminescence was done. The result showed that the nanowires formed had only gallium and oxygen content, whereas their nature was amorphous. Wires synthesized by this process had a diameter of between 40–110 nm; and its photoluminescence showed that it had two bands of emissions, of which one was weaker and the other stronger. The weaker one was at 365 nm, and the stronger one was at 470 nm; these correspond to the intrinsic transition and vacancies in gallium oxide, respectively.

Two years later, two of the previous four researchers [130] used the MOCVD synthesis technique to produce nanowires of gallium oxide. The substrate was silicon (100). The process started with the cleaning of the substrate, which was done with acetone. Trimethylgallium was used for the gallium source to deposit on the substrate, and oxygen was also used. The flow rate of oxygen was 6 sccm and argon carrier gas was set at 30 sccm. The substrate temperature was elevated to 650 °C and the times allowed for deposition were 3, 4 and 5 min. When the structural characterization was done on the prepared samples using SEM, TEM, and XRD, it was found that deposition time affected the accumulation of the nanostructures. Nanowires formed were denser and thicker at the highest deposition of 5 min, when compared to the other two. The XRD data illustrated that the nanowires had an amorphous nature, and the TEM showed that the nanowires consisted of only gallium and oxygen content. 

**Figure 7:**
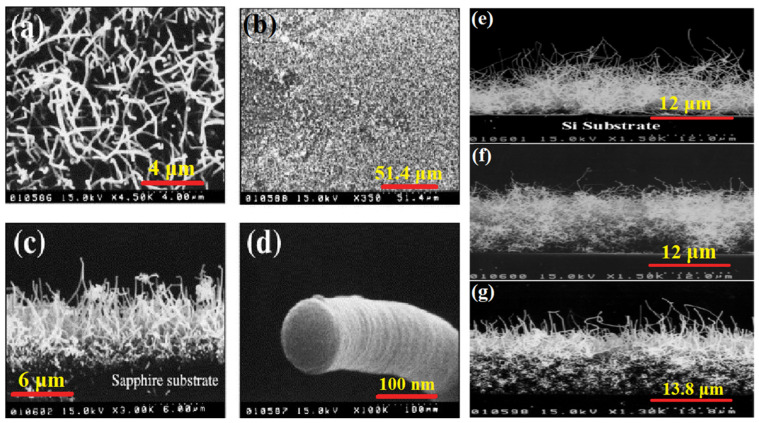
The four images (**a**–**d**) show various views in the SEM imaging of the sample with the nanowires visible [129]. “Reprinted with permission from Ref. [129]. Copyright (2004), Elsevier”. (**e**–**g**) show the three images of samples with different deposition times of 3, 4 and 5 min [130]. “Reprinted with permission from Ref. [130]. Copyright (2006), Taylor and Francis Online”.

As the Table 3 and Figure 7 make clear, all the processes produced nanowires with no impurities. However, the growth of the nanowires was not channelized and was still random. Here, the research with the catalyst showed that adding a catalyst allowed 2D nanostructures along with 1D nanostructures, with increasing deposition time.

The work done in [131] reported that the formation of nanowires was linked with the vapor liquid and vapor solid mechanism. However, the work done in [129,130] reported that nanowire synthesis did not require a catalyst, as the entire process was not affected by the VS and VL state. The deposition rates were faster without the catalyst and similar growth patterns could be observed in these works. 

### 3.4. Pulsed Laser Deposition

Pulsed Laser Deposition is a cheap and easy technique to synthesize nanoparticles [132,133,134,135,136,137]. The laser ablates the target, and the deposition takes place on a substrate. The process is generally performed in a chamber filled with various gases under constant pressure. The target and substrate are separated or placed apart from each other. This technique is very effective for thin films of gallium oxide. Gallium oxide thin films were produced using a ceramic gallium oxide, targeted at 99.99% purity, and the substrate that was used to deposit the nanoparticles was a c-plane sapphire with an (0001) orientation [138]. A KrF laser was employed for the ablation, and the temperature of the sapphire was varied between 600 and 1000 °C (400, 600, 800, 1000). The distance between the substrate and the target was small and was maintained at 50 mm. It was observed that as the temperature increased, the crystalline formation was better, and a monoclinic crystalline structure could be achieved. The thickness of the structure was measured to be 220 nm, and the materials synthesized were compatible for deep UV application. Transmittance data showed a gradual blue shift, and band gaps (4.65, 4.86, 4.92, 4.96) of the samples were calculated by using the Tauc plot method, while absorption was also determined. It was observed that the films at higher temperatures had fewer defects and vacancies. Yu et al. produced photodetectors using the films produced at 600 and 800 °C. The IV curve analysis was performed in a dark environment to analyze the dark current measurement and showed that synthesis of the materials at 800 °C was more effective, as they had better structure and fewer vacancies of oxygen. In another instance where high quality crystalline structure was required, pulse laser deposition was employed, as gallium oxide is temperature and chemical sensitive material. In 2016, Garten et al. were able to synthesize gallium oxide films on two substrates, gallium oxide and c-plane sapphire [139]. The target was Sn-doped gallium oxide with doping of 2%. This synthesis was performed using a KrF laser. The oxygen pressure was maintained at a constant, and temperature for gallium oxide on the sapphire substrate was varied along with the frequency. Temperature was varied from 200 to 600 °C, approximately; and it was observed that a small variation in the temperature could affect the structure of the thin film. At a lower temperature, or in a cooler region, the material was amorphous in nature, and at a higher temperature crystalline growth was noted, with β-Ga_2_O_3_ formation at a temperature of 500 °C. Additionally, the transmittance data showed a change in conductivity. It was noted that even with Sn doping, the amorphous gallium oxide structures had a lower frequency than the others. The crystalline structure was analyzed using XRD, and this work produced crystalline structures at a lower temperature than conventional techniques.

One of the earlier instances of synthesized nanomaterials using the laser deposition method was demonstrated in 2004 [140]. The team involved in the study used GaN as the target material and p-type silicon with a 100 orientation as the substrate. Initially, a vacuum was maintained in the chamber, and then N_2_ gas was passed into the chamber while maintaining pressure between 1-100 Torr. The target was rotated to have complete ablation. The laser used was a krypton fluoride laser, which is a type of excimer laser usually employed in medicine to correct the refractive index of an eye to eliminate myopia. In this synthesis, the substrate and target were placed 1 to 3 cm apart. The synthesis was done at a wavelength of 248 nm with a pulse of energy between 100 and 200 mJ. The parameter that changed here was the pressure of the chamber. Monoclinic nanoparticle formation was confirmed using TEM analysis. The nanochain formation was also seen through HRTEM; these chains were formed due to the amalgamation of spherical nanoparticles. The presence of Ga and O_2_ was observed using EDX. The pressure in the chamber contributed to the change in the diameter of the nanoparticles. As the pressure increased, the diameter of the nanoparticles decreased. This effect could be due to the size confinement. Also, the emission of blue light in the photoluminescence was seen, and the material could be utilized for the production of FET.

**Figure 8:**
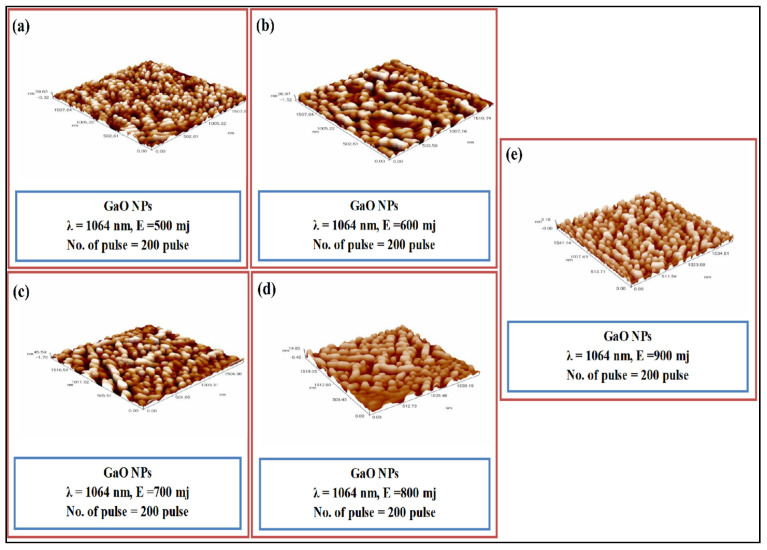
The AFM of the nanoparticles produced by pulsed laser deposition. (**a**) 500 mJ; (**b**) 600 mJ; (**c**) 700 mJ; (**d**) 800 mJ; (**e**) 900 mJ [141]. “Reprinted with permission from Ref. [141]. Copyright (2020), College of Science, University of Baghdad”.

Pulse laser deposition is mostly employed in the fabrication of thin films. However, recently, many researchers have been able to synthesize nanowires using this technique with a temperature greater than 500 °C. In 2012, Hameed et al. [141] used gallium oxide powder, instead of GaN. The powder was converted into pellets and then the deposition was done on a glass substrate. The glass substrate was properly cleaned. The deposition was performed at room temperature based on the parameters defined in the table. The energy of the laser was varied in this process from 500 to 900 mJ, while keeping the other parameters constant. Based on the AFM images in Figure 8, it can be seen that nanoparticles were synthesized and, also, with increasing laser energy the thickness of the nanoparticles increased as well.

**Figure 9:**
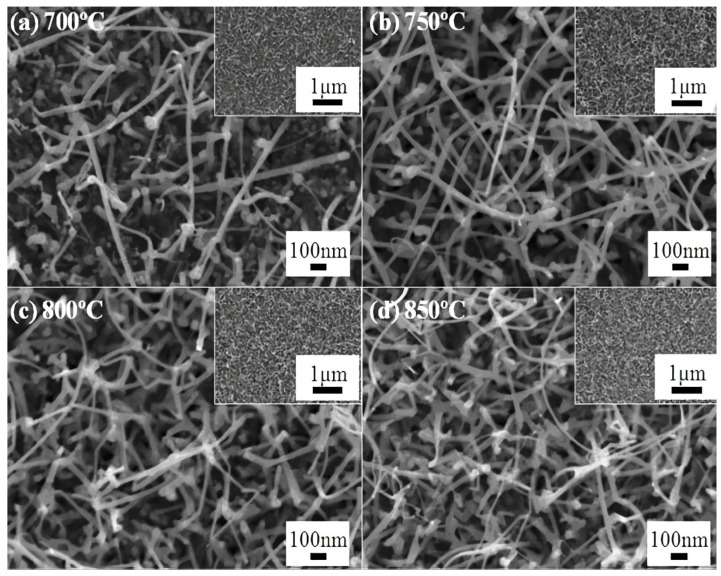
FE-SEM images of gallium oxide nanowires synthesized by pulse laser deposition using gold catalyst with varying temperatures. (**a**) 700 °C; (**b**) 800 °C; (**c**) 850 °C; (**d**) 900 °C [142]. “Reprinted with permission from Ref. [142]. Copyright (2012), Scientific Research”.

In more recent research results [142], the researchers used a catalyst for the synthesis of gallium oxide nanowires using the pulse laser deposition method. The employed catalyst was gold and it was sputtered on the aluminum oxide substrate, which was then used for laser deposition of gallium oxide nanowires using sintered pellets of beta gallium oxide. The substrate temperature was varied here from 700 to 850 °C. SEM images of the samples, with 3 nm thickness of gold, reported that the formation of nanowires was based on the sputtered growth. The nanoparticles were very much less where the surface roughness was higher, and in the region with lesser thickness the nanowire density was much less. This was because in the region which was far from the edge there were larger gold particles and less annealing, due to which the vapor-liquid-solid process was not very active. This shows that the density of the particles increased in the region where the gold particles were more annealed. As can be seen in Figure 9, for various temperatures of the process the density of the nanowires was higher for the higher temperatures.

The research works have all noted that the synthesis process is temperature and pressure sensitive, and a slight variation can be used to tune the properties. The process is simple, and the desired structure of gallium oxide nanoparticles can be synthesized. At higher pressure and temperature, researchers have observed the formation of a crystalline structure, and at lower temperature and pressure, an amorphous structure is obtained. Employing a catalyst helps in the synthesis of 1D nanostructures and varying the amount of the catalyst can result in the formation of different sizes of nanowires. It was observed that regions with gold had higher concentrations of nanowires, which shows the various morphologies of the formed nanowires.

### 3.5. Molecular Beam Epitaxy

Molecular Beam Epitaxy (MBE) is another technique employed for the synthesis of nanomaterials [2,143,144]. It is a widely used technique, as it produces the best thin films. This process occurs in a high vacuum chamber, which has a complex component structure. The process requires the use of effusion cells and the metals are heated and deposited on the substrate. This process allows a changing orientation of the thin films, as it uses a motor that can rotate the substrate. It employs a RHEED (Reflection High Energy Electron Diffraction) gun, which is used as a tool to check for the surface morphology of the material that is being formed. The overall process is one of the most complex and expensive [145].

A study conducted in 2010 [146] prepared gallium oxide thin films using the MBE technique assisted with plasma. The team produced two differently grown crystalline structures, one on a sapphire substrate and the other on a gallium oxide substrate. The pressure of the plasma beam was varied, keeping the oxygen pressure in the chamber constant. The material was heated up to 700 °C in the deposition, and the RHEED gun noted the growth characteristics. It was observed by the authors that the pressure of the plasma beam played an important role in the rate at which growth happened. A low pressure indicated higher growth, and vice versa. This effect was due to the formation of the gases in the chamber as the pressure rose.

Ghose et al. prepared beta gallium oxide thin films using the MBE technique. They also used oxygen plasma for the process and chose c-plane sapphire as the substrate [147]. Here, gallium in the elemental form and beta gallium oxide were used for deposition. The resultant nanoparticles showed a higher band gap of a 5.02 eV maximum for one of the samples, which was higher than the 4.5–4.9 eV presented by various authors [9,136,148,149,150]. Also, the prepared samples were of higher quality and smoothness and showed better optical properties. The temperature of the substrate was an important factor that determined the formation layer and the quality of the thin film.

**Figure 10:**
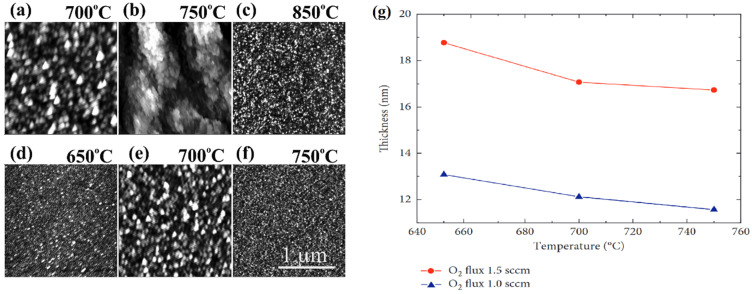
The AFM of the gallium oxide thin films synthesized by MBE by varying the growth temperature (**a**–**f**) 650-850 °C [147]. “Reprinted with permission from Ref. [147]. Copyright (2016), AIP Publishing”. (**g**) variation in the thickness of two different samples prepared by varying oxygen flux over the temperature range [151]. “Reprinted with permission from Ref. [151]. Copyright (2018), Hindawi”.

Subsequently, the formation of gallium oxide crystalline structure was demonstrated on sapphire substrate using plasma assisted MBE [151]. The growth temperature was varied between 650 and 750 °C. Due to the varying temperature, the surface roughness decreased and crystallinity of the thin films increased. This was due to the mismatch of the lattice between the substrate and the gallium oxide film. The size of the grains reduced as well. It was also noted that, along with the temperature, variation in the oxygen flux could affect the sample thickness and roughness. Figure 10b shows the graph plotted for the two oxygen fluxes for varying temperature against the thickness of the samples. When higher oxygen flux was used, the thickness would be higher and the roughness would decrease on the substrate.

The nanomaterials that were prepared using this synthesis process had the best nanostructure quality, which depended on various parameters, such as the pressure of the oxygen, flow rate of oxygen, temperature of the substrate, and the formation of gases in the chamber. Varying these parameters would allow the researchers to tune the properties of the nanostructures, along with the structure or layer formation of the thin films.

## 4. Properties of Gallium Oxide

**Figure 11:**
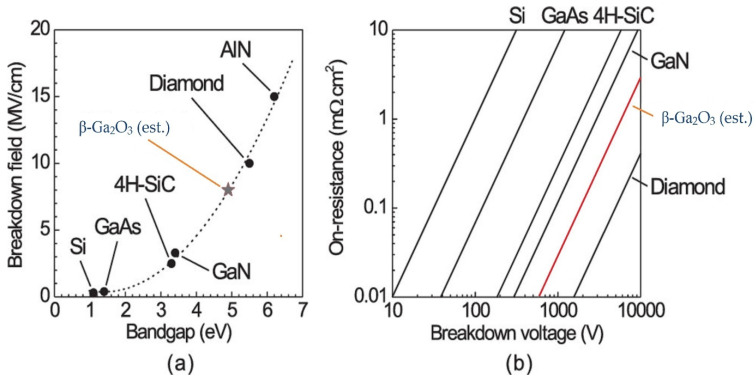
Comparison of breakdown field of various semiconductors (**a**,**b**) [1]. “Reprinted with permission from Ref. [1]. Copyright (2013), AIP Publishing”.

Physical and optical properties of gallium oxide nanostructures (Table 4) are highly dependent on the morphology or the structure of the nanostructures (composition and crystallization). Controlling these properties can be achieved by synthesis method, fabrication parameters and post-processing technique, as mentioned earlier in various fabrication techniques. For example, one of the major parameters is the annealing temperature. Many techniques that are used to synthesize Ga_2_O_3_ nanostructures employ annealing, and annealing helps in the formation of better nanostructures and facilitates the formation of beta structure gallium oxide [57]. As has been discussed, the beta phase is the only stable phase for gallium oxide and, thus, most work has been carried out in that phase. Also, the availability of beta phase makes it more popular among all the other phases of Ga_2_O_3_. The major highlight among all the properties of gallium oxide is its critical breakdown electric field, which is very much higher than others, like GaN (~5 MV/cm) and SiC (~3 MV/cm), as can be seen in Figure 11, along with low electron mobility.

### 4.1. Optical Properties

Gallium has a high band gap, as already discussed. The optical properties of nanostructures depend on both material/chemical properties and surface topology [161].

#### 4.1.1. Band Gap

**Figure 12:**
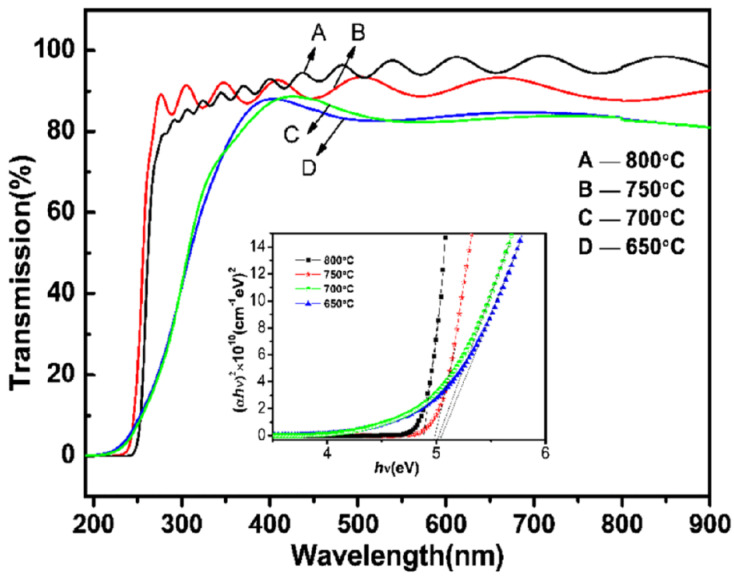
The band gap graph, plotted for beta gallium oxide at different temperatures grown using MOCVD. The graph is plotted using the Tauc plot theory [170]. “Reprinted with permission from Ref. [170]. Copyright (2017), SpringerLink”.

Band gap defines one of the most important optical properties of gallium oxide. The band gap of gallium oxide is high, and with the formation of gallium oxide nanostructures, it was noticed that there could be a slight variation in the band gap [162]. It could be noticed that the nanowires of gallium oxide show better properties than other nanostructures, as they have fewer defects. This quality is due to a lower mismatch been the lattice of the substrate and the thin film that grows [163]. The amount of strain in the nanowires is negligible. One of the most common approaches for determining the band gap of nanomaterials is light spectroscopy and calculating the transmittance, absorbance or reflectance of the nanomaterial. Using either Tauc’s plot [164,165,166] or the Kubelka-Munk function [131,167,168], a graph is plotted against photon energy (eV) by which the band gap is determined, as can be seen in Figure 12. When nanostructures of gallium oxide are synthesized, it can be noted that the band gap of the material increases. This increase is attributed to the structure of the thin films and is related to the change in the state of the conduction band [169]. 

**Figure 13:**
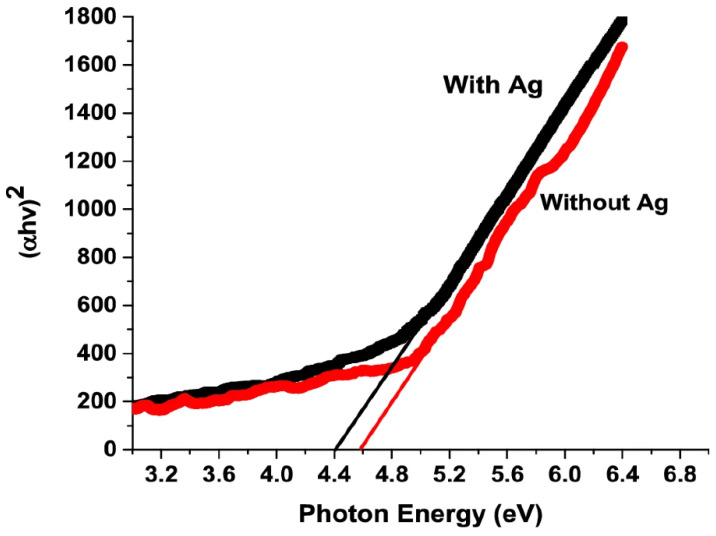
Band gap of gallium oxide nanowires using an Ag catalyst [171]. “Reprinted with permission from Ref. [171]. Copyright (2020), nature”.

In 2020, it was documented (Figure 13) that the band gap of gallium oxide nanostructures can be affected by a catalyst during the synthesis [171]. The previous work on this method was also highlighted, and it showed that different catalysts could affect the band gap (Table 5). In the study, the team compared two samples of gallium oxide, one using 5 nm of Ag (silver) and the other without it, and it was observed that the one with Ag had a band gap of 4.4 eV and the other had a band gap of 4.6 eV. This difference was due to the addition of a catalyst. It was also illustrated that other catalysts had different effects on the band gap. It can be viewed from the table that, using a metal catalyst, the band gap of the nanostructures of gallium oxide decreases. Also, from Figure 13 it is clear that the nanowires that were synthesized without using the Ag catalyst had a higher band gap. This is because adding Ag would enhance the conductivity of the material and, thus, decrease the band gap of gallium oxide. In another research work [27], the band gap of the synthesized nanostructures could increase if the structures were prepared in an environment with adequate nitrogen flow rate in the CVD process.

#### 4.1.2. Photoluminescence (PL)

Photoluminescence occurs in various emission bands, such as red, blue, and green, at the band gap of the nanostructures of gallium oxide. This technique generally employs irradiation using a laser beam, and the light generated by the nanomaterial is then collected. Based on the spectrum of the luminescence, the presence of any impurity in the nanomaterial is determined. This process involves exciting the material to move its electrons to a higher electronic state, after which they then move back to a lower energy level. Along with impurities, it also determines the crystallinity of the structure and determines the presence of any disorder. The photons of the material are excited above the band gap of the material.

**Figure 14:**
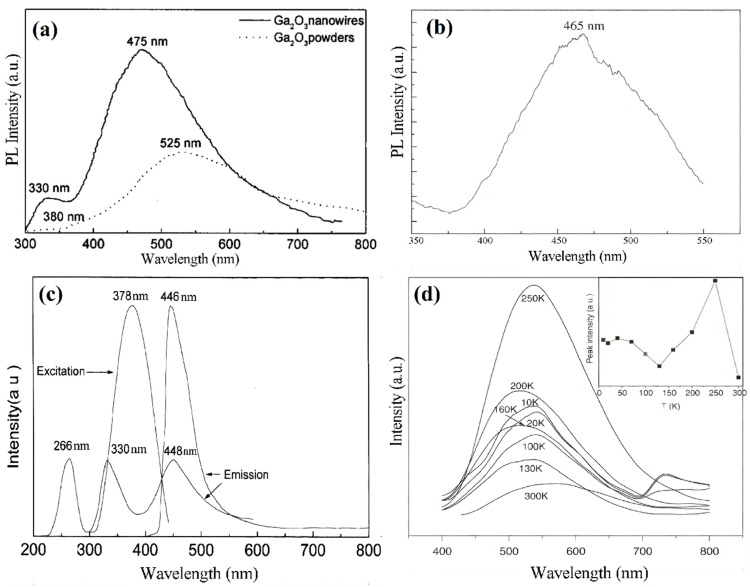
The photoluminescence of gallium oxide nanostructures (**a**) blue emission peak at 250 nm excitation [172]. “Reprinted with permission from Ref. [172]. Copyright (2001), AIP Publishing”. (**b**) blue emission peak 250 nm excitation [173]. “Reprinted with permission from Ref. [173]. Copyright (2005), Elsevier”. (**c**) blue emission at 446 nm and UV emission at 330 nm [180]. “Reprinted with permission from Ref. [180]. Copyright (2000), Elsevier”. (**d**) green emission at 10 K and 300 K temperature excitation [181]. “Reprinted with permission from Ref. [181]. Copyright (2006), Elsevier”.

Over the course of time, researchers have observed that the beta structure of gallium oxide has various photoluminescence emissions, among which UV emission is the most prevalent. The other emissions that have been recorded are blue and green. The blue emissions are generally found by researchers to be due to the presence of oxygen vacancies [172,173,174,175]. Green emissions have been reported due to extended defects and some impurities, that include Sn, Be and Ge [174,176,177,178,179]. One research also reported green emission in gallium oxide nanowires due to Cr and Er doping [176]. Figure 14 shows the various photoluminescence emission peaks observed from the nanostructures of gallium oxide.

Further, photoluminescence on crystalline gallium oxide at room temperature was demonstrated by Wu et al. [180], and the group was able to note an emission of blue light on excitation to 378 nm, and the peak was observed at 446 nm (Figure 14c,d). The property here was closer to the single crystal gallium oxide, and they also observed the emission of UV, which was at 330 nm. The Stokes shift, however, was much lower than that of a single crystal, which was explained by the size confinement associated with low Stokes shift. This effect was due to the trapping of the donor electron and the acceptor electron. The intensity of the blue light would decrease with an increase in temperature, as an increase in temperature would release the holes and electrons of the acceptor and donor. This property allows the material to have useful applications in the opto-electric field. [175] also reviewed various researchers and mentioned that the excitation of the acceptor and the donor leads to the generation of a hole and an electron, respectively, leading to trapping and then emission of blue light.

### 4.2. Electrical Properties

#### Photocurrent and Dark Current

Among the electrical properties of gallium oxide, this is one of the most important, as it defines the ability of the semiconductor to work as a diode and allows measurement of the current generated by incident light or photon energy. The semiconductor’s morphology plays an important role in defining these properties. Photocurrent is the current obtained from the semiconductor when photon energy, or a light source, is applied, and due to which the electrons and holes move towards the anode and cathode, respectively. This movement results in the formation of the current, which is the photocurrent. The dark current here is the current that is measured when there is no photon energy applied to the semiconductor. For the application of a semiconductor as a photodiode, the dark current is a hindrance and should be low.

An experiment published in Nanoscale 2011 [182] evaluated the properties of gallium oxide nanobelts using the two-probe method, where the nanobelts were connected to Cr/Au electrodes, photon energy was applied at various wavelengths, and the photocurrent plotted. The study remarked on the sensitivity of nanobelts to light energy at lower wavelengths below 300 nm. This responsivity of the nanobelt decreases at higher wavelengths. The photocurrent was shown to be produced at an energy higher than that of the band gap and, thus, was claimed to be due to the electron-hole pairs. Also, at normal room pressure in air, the dark current was higher than that of the photocurrent, and in a vacuum, the photocurrent was higher, due to the lack of oxygen on the nanobelts.

## 5. Applications

Applications relating to gallium oxide nanostructures are increasing, and with more researchers taking an interest in the field, more applications are being reported. There are still many challenges to the application of gallium oxide nanostructures, such as environmental considerations, and the cost-effectiveness of the methods that are employed to produce the nanostructures.

### 5.1. Gas Sensing Applications

**Figure 15:**
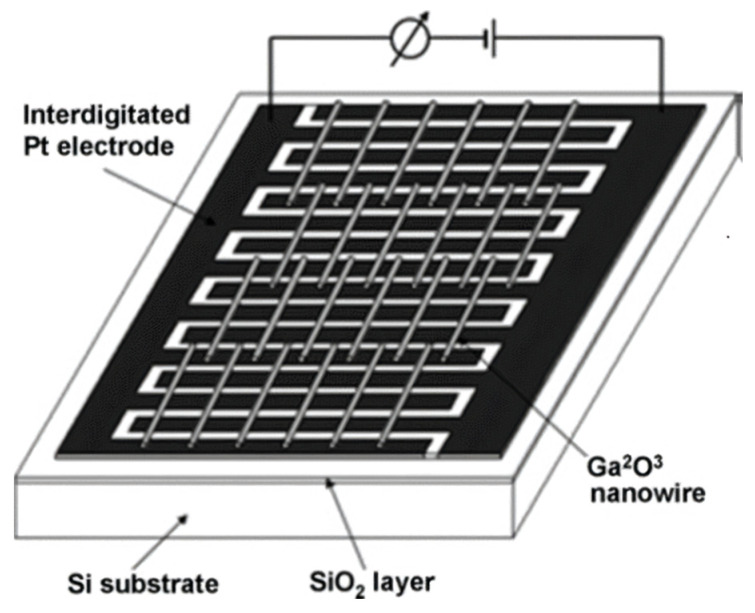
The gas sensor made from Ga_2_O_3_ nanowires [185]. “Reprinted with permission from Ref. [185]. Copyright (2008), Elsevier”.

Gallium oxide nanomaterials are a promising candidate for gas sensing applications. Researchers have always shown a keen interest in these applications for gallium oxide, as the atmospheric composition affects the conductivity of Ga_2_O_3_ at increased temperatures [183]. A number of researches have focused on various gas sensors as one of the models is demonstrated in Figure 15. [175,184,185,186].

In 2007, Bartic et al. [27] made resistive oxygen sensors that were designed for higher temperature oxygen sensing. The sensor was designed using a single crystal gallium oxide and platinum interdigital electrode sandwich. These sensors were helpful for sensing oxygen in the atmosphere at a temperature of 1000 °C. The samples were prepared with two different techniques, namely, Rf sputtering and chemical deposition. The results showed that the preparation technique affected the properties. The response time played an important role, and it was efficient to keep a short response time. In this study, it was clearly observed that the samples with Rf sputtering had better sensitivity, but the chemical deposition samples, on the other hand, could be made more cheaply with similar characteristics.

**Figure 16:**
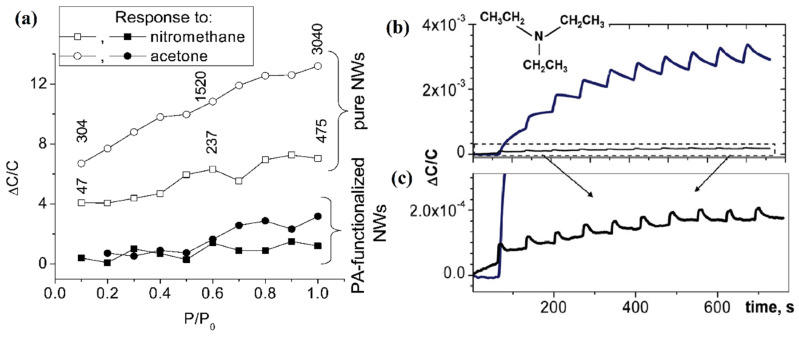
Graphs showing response of gallium oxide nanowires and PA adsorbed gallium oxide nanowires. The reduction in the ppm of nitromethane and acetone is clearly visible. (**a**) shows the response to TEA by pure nanowires and (**b**,**c**) shows the response by PA adsorbed nanowires [187]. “Reprinted with permission from Ref. [187]. Copyright (2010), Elsevier”.

In the same year, a group was able to synthesize gallium oxide nanowires using chemical thermal evaporation and utilized the technology to manufacture gas sensors [185]. The sensors were prepared as shown in Figure 16, using nanowires, Si as substrate, and a Pt electrode. The gas sensors showed a greater response for O_2_ and CO in a temperature range between 100 to 500 °C. The sensors that were previously made showed responses at very high temperatures above 900 °C, whereas the gas sensors showed a peak at 300 °C for oxygen and a peak at 200 °C for carbon monoxide.

Interesting research work was done by Mazeina [187], where gas sensors were based on capacitance and instead of sensing at higher temperature, they were effective at room temperature (Figure 16). The group was able to overcome the disadvantage of the previously reported gas sensors, which were not able to be selective to common hydrocarbons like Acetone and nitromethane. They overcame the disadvantages by adsorbing a layer of pyruvic acid (PA) onto gallium oxide nanowires. Also, they were able to increase the sensing response to triethylamine (TEA).

### 5.2. Photovoltaic Devices

The application of gallium oxide in photovoltaics is highly researched; as, with the wide gap, the nanostructures have higher optical properties, as discussed in this paper. The nanostructures are employed in solar-blind photodetectors and are vigorously researched for higher sensitivity and response time [47,92,182,188]. The research initially focused only on using the crystalline structure of the gallium oxide nanostructures. The importance of β-Ga_2_O_3_ increased as researchers focused on maintaining the crystalline phase, as it shows better properties and is more stable and easier to prepare [162,189]. However, studies on the amorphous phase of gallium oxide indicated that it also allows for some prominent applications, as mentioned by Qian et al. [190].

The synthesis of ε-Ga_2_O_3_ nanostructures was followed by several tests to report on their application as solar blind photodetectors. Electrical-optical results showed that one design showed a great response, and the material was stable for a varied bias [191]. The photocurrent results showed that the conductance of the material increased with the increase in the temperature. The measurements under light and dark conditions displayed that ε-Ga_2_O_3_ showed a great response over longer periods, and noise was also minimal for the light condition. This property of the material showed greater resistance to a dark condition, which is of great relevance for solar blind photodetector application.

Qian et al. used the amorphous nanostructures of gallium oxide to produce solar blind photodetectors, based on the work of a previous researcher. If a thin layer of amorphous gallium oxide nanostructures is synthesized through pulse laser deposition at lower temperature and with oxygen, then there is a defect band formation that makes the material oxygen-deficient [192]. Qian et al. prepared photodetectors using amorphous gallium oxide, which showed better responsivity (70.26 A/W) than the β-Ga_2_O_3_ photodetector sample (4.21 A/W). This difference was due to the defect band, which was observed by surface morphology.

### 5.3. Higher Power Devices

**Figure 17:**
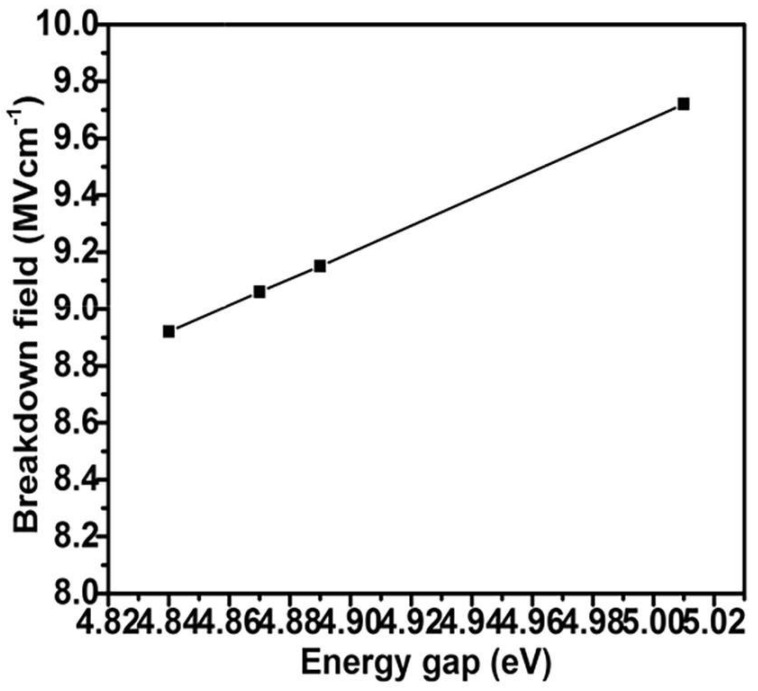
The plotted graph of enhanced E_c_ for beta gallium oxide microstructures [194]. “Reprinted with permission from Ref. [194]. Copyright (2019), Elsevier”.

The wide band gap of gallium oxide, as discussed previously, makes it a promising material for high-power applications. For high-power application use of gallium oxide, modulation of conductance is required, which can be achieved through the gate voltage of a Field Effect Transistor (FET) [193]. The performance of gallium oxide in high-power devices surpasses all other semiconductor materials, like Si, SiC, GaN. This is due to high critical field strength along with a higher band gap. This feature allows the material to have lower losses and gallium oxide can withstand higher temperatures. Rex et al. in their work prepared micro-sized structures of beta gallium oxide using hydrogen-assisted CVD and were able to achieve a higher critical field value between 8.9-9.7 MV/cm (Figure 17) by varying the deposition temperature [194].

Another factor that defines the usage of gallium oxide in higher power devices is Baliga’s Figure of Merit (BFOM) [55,195,196].

BFOM ∝ εμE_c_^2^ (1)

The BFOM is directly proportional to the critical electrical field value of the material. The only concern with gallium in higher power devices is its lower thermal conductivity [2,18,197], which is comparatively far lower than the aforementioned materials.

The research on the application of gallium oxide as a field-effect transistor was not very great, and practical use was not demonstrated much before 2012. Ref. [1] showed the result and potential for the potential use of gallium oxide as FET or higher power device application. They prepared samples of MESFET from gallium oxide using Molecular Beam Epitaxy, and in one of the samples, reactive ion etching was also done. This result showed that the etching process created oxygen vacancies in the material, causing defects on the surface, and, due to these defects, the samples reported ohmic behavior.

In 2012, gallium oxide nanowires were put into use to amplify the efficiency of the transport properties of transistors [198]. These transistors had reduced gate length from 500 to 50 nm. The aim was to have positive charge polarization, which would be a result of the size confinement because of a high dielectric constant, which is a property of gallium oxide nanowires. This study resulted in containing the leakage of any charge and provided a better transport channel, which helped in scaling the device and maintaining the working of the transistor.

Another application of gallium oxide nanowires was journaled in 2005 by Chang et al. [199]. In this study, gallium oxide was further doped with zinc for the application of FET. Electron transfer studies were done on the doped sample. It was observed that due to the doping of the gallium nanowires, the source drain voltage increased. The current decreased at the positive gate voltage and vice versa for the sample, which was due to the increase in the number of holes by doping. The semiconductor subsequently exhibited p-type properties.

## 6. Conclusions

In this review, we have discussed the synthesis of beta gallium oxide nanostructures, along with their properties, and focused attention on the major applications of gallium oxide.

Gallium oxide nanostructures have shown a potential development in emerging technologies based on varied nanostructures. These structures are influenced by various techniques employed in their synthesis. The parameters used also play a major role, as with change in parameters a change in formation was observed. Studies have noted a wide band gap and shown potential applications. Processes used for the fabrication are expensive, and there is a requirement for a cheaper method. Processes in discussion are relatively complex, hence, mass production of the material will increase the expenses. The properties of materials have been enhanced as size was reduced to nanoscale and its photoluminescence properties showed that nanostructured beta gallium oxide offers a wider range of emissions.

The current research on gallium oxide nanomaterials is at a phase where the studies are based on different methodologies and finding a suitable property for suitable applications. Polymorphs of gallium oxide nanostructures are yet to be completely explored, and a major focus on β-Ga_2_O_3_, which is monoclinic, has been observed. There has been an interest in other structures as well, which could be a potential aspect for future studies. Also, the applications are mostly focused on gas sensors, where the researchers have shown that oxygen vacancies in the gallium oxide nanomaterial have a vital role in these applications. Many sensors relating to CO and oxygen were fabricated and tested as well. However, gallium oxide has shown capability of use in other applications as well, which could replace other wide band gap semiconductors. One of these possibilities is its being employed in high-power devices. Even though gallium oxide has poor conductivity, due to a large band gap, its ability to handle large power allows it to work in many higher power devices. Low thermal conductivity and p-type doping is still a challenge, although some advances have been made in the latter.

## Figures and Tables

**Table 1:** Polymorphs of Gallium Oxide.

Structure		Comments	References
**α-Ga_2_O_3_**	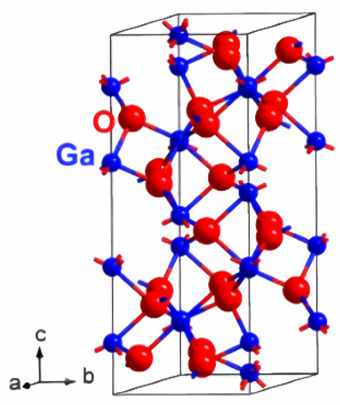	This structure is another common structure, apart from beta gallium oxide. It has a similar corundum structure to Al_2_O_3_. Making fine crystals is a difficult job. The α-Ga_2_O_3_ structure can be maintained only at around 550 °C and above that a phase transformation to β-Ga_2_O_3_ takes place.	[18,51,53]
**β-Ga_2_O_3_**	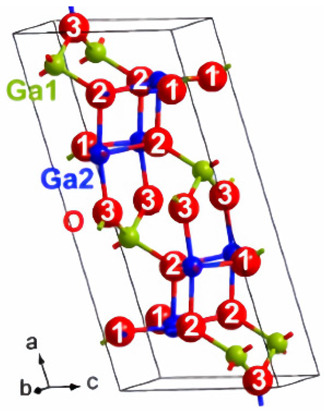	It is the most stable of all in ambient condition and has major interest from researchers and, as already mentioned, it has a monoclinic structure with parameters a = 12.19 A, b = 3.016 A, c = 5.80 A β = 103.7^0^	[54,55,56]
**γ-Ga_2_O_3_**	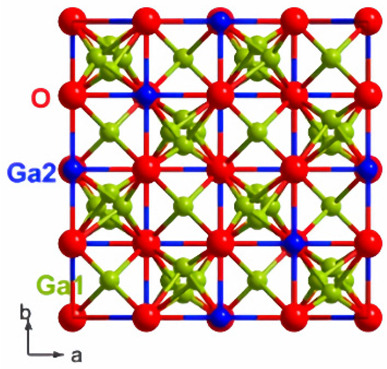	The preparation of this polymorph is simple as it just requires the oxidation of gallium in amino alcohol, like ethanolamine.	[57,58]
**ε-Ga_2_O_3_**	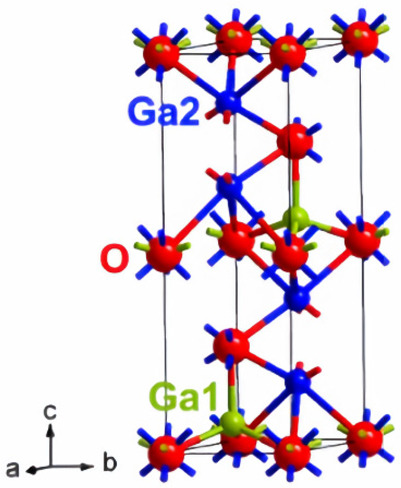	This structure of gallium oxide can be metastable at higher pressure conditions. Also, upon heating it can transform to alpha and beta phases. Furthermore, it exhibits ferroelectric property.	[59,60]
**δ-Ga_2_O_3_**	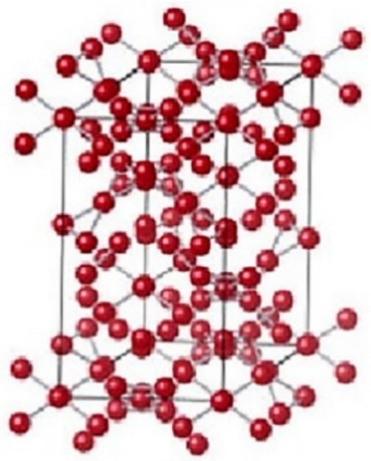	This structure was initially thought to be a phase which was similar to ε-Ga_2_O_3_. The structure was presumed to be a nano-crystal form of ε-Ga_2_O_3_. However, later it was confirmed that it is not a nanostructure or another phase and is a different cubic structure.	[61,62]

**Table 2:** Nanostructures of beta gallium oxide based on various parameters of magnetron sputtering.

Ref.	Substrate	Gas	Chamber Pressure, Pa	RF Power, W	Substrate Temperature, °C	Annealing Temperature, °C	Comments
[100]	Si (111)	Ar (99.999%)	2 Pa	150	Room temperature (RT)	850, 900, 950, 100 in Ammonia	Here, single crystal nanorods were synthesized and variation in the annealing temperature affected the morphology.
[101]	SiN/Si (001), SiO_x_/Si (001), Glass (EAGLE 2000)	Ar (99.999%)	0.6666	100	RT to 625		The vapor liquid phase mechanism worked for higher temperature and oxygen deficient conditions for the formation of nanowires with higher crystallinity.
[102]	Quartz and Si (100)	Ar and O_2_	0.6666	50	RT	600, 800, 950, 1000 in air	Both substrates had a similar result for film, thickness, surface roughness, density and deposition times. The major factor here was the annealing temperature.

**Table 3:** Nanostructures of beta gallium oxide, based on various parameters of CVD.

	Substrate	Carrier Gas	Sources	Catalyst	Deposition Time (mins)	Tamperature	Comments
[129]	Sapphire	Argon	Ga—TMGa O_2_—Oxygen		5	600 °C	Nanowire formation was observed and also, they were amorphous in nature. A catalyst was not used and yet still there was synthesis of nanowires without impurities.
[130]	Si (100)	Argon	Ga—TMGa O_2_—Oxygen		3, 4 and 5	650 °C	Varying the deposition times allowed a change in the density of the formed wires, which confirmed that deposition time affects the growth of nanowires.
[131]	Sapphire	Nitrogen	Ga—Gallium metal O_2_—water vapor	Nickel	30, 60, 90	900 °C	The adding of a catalyst helped in attaining nanowires of monoclinic gallium oxide structure. The varying deposition time also showed that nanosheets were synthesized as well.

**Table 4:** Common properties of β-Ga_2_O_3_.

Properties	Value	References
Breakdown Electric field, E_c_	8 MV/cm	[75,152,153]
Thermal Conductivity	0.1–0.3 W/cm-K @ RT	[154,155]
Mobility	100 cm^2^/Vs	[156,157]
Lattice parameters	A = 12.19 A, b = 3.016 A, c = 5.80 A, β = 103.7^o^	[54,55]
Space group	C2/m	[49,50]
Melting Point	1793 °C	[70,158]
Refractive Index	1.95 to 2.1	[159,160]
Band Gap	~4.9 eV	[161,162,163,164]

**Table 5:** Band gap using different catalysts, [171]. “Reprinted with permission from Ref. [171]. Copyright (2020), nature”.

Catalyst	Band Gap (eV)
Ni	4.30
Au	4.7–4.8
No Catalyst	4.56–4.6
Ag	4.4

## Data Availability

The data will be provided upon request.
